# Sentence-based mental simulations: Evidence from behavioral experiments using garden-path sentences

**DOI:** 10.3758/s13421-022-01367-2

**Published:** 2022-10-28

**Authors:** Emanuel Schütt, Carolin Dudschig, Benjamin K. Bergen, Barbara Kaup

**Affiliations:** 1 Department of Psychology, Language and Cognition Research Group, University of Tübingen, Schleichstr. 4, 72076 Tübingen, Germany; 2 Department of Cognitive Science, University of California at San Diego, La Jolla, CA USA

**Keywords:** Sentence comprehension, Embodied cognition, Mental simulations, Garden-path sentences, Incrementality

## Abstract

Language comprehenders activate mental representations of sensorimotor experiences related to the content of utterances they process. However, it is still unclear whether these sensorimotor simulations are driven by associations with words or by a more complex process of meaning composition into larger linguistic expressions, such as sentences. In two experiments, we investigated whether comprehenders indeed create sentence-based simulations. Materials were constructed such that simulation effects could only emerge from sentence meaning and not from word-based associations alone. We additionally asked when during sentence processing these simulations are constructed, using a garden-path paradigm. Participants read either a garden-path sentence (e.g., “As Mary ate the egg was in the fridge”) or a corresponding unambiguous control with the same meaning and words (e.g., “The egg was in the fridge as Mary ate”). Participants then judged whether a depicted entity was mentioned in the sentence or not. In both experiments, picture response times were faster when the picture was compatible (vs. incompatible) with the sentence-based interpretation of the target entity (e.g., both for garden-path and control sentence: an unpeeled egg), suggesting that participants created simulations based on the sentence content and only operating over the sentence as a whole.

Throughout the past two decades, the embodied cognition view has had an increasing influence on research concerned with human cognition (e.g., Chatterjee, 2010). This is especially true for the area of language comprehension. Embodied cognition views of human language comprehension (e.g., Barsalou, 1999; Bergen, 2012; Glenberg & Kaschak, 2002; Zwaan & Madden, 2005) propose that comprehenders grasp the meaning of a word by mentally simulating the word’s referent. Concretely, upon hearing or reading a word, comprehension is effected by reactivating sensorimotor experiences that are associated with its referent. For example, when hearing the word “sky”, comprehenders might reactivate the experience of perceiving the typical color of the sky (i.e., blue) or the experience of looking up. Importantly, words are hypothesized to activate such sensorimotor experiences as—particularly during childhood—they tend to regularly co-occur with their referents in everyday life (Vogt et al., 2019). For instance, a mother may point at a cat and say to her child: “Look! A cat”.

Naturally, words are typically encountered together with other words forming phrases and sentences that convey meaning beyond the word level. Embodied cognition views of language comprehension suggest that comprehenders combine reactivated word-based sensorimotor experiences to create mental simulations corresponding to the meaning of the phrase or sentence in question. Thus, the literature proposes two types of simulation mechanism: Word-based simulations that are sensorimotor experiences triggered by individual words and sentence-based simulations that result from merging word-based simulations to obtain a combined meaning on phrasal or sentential level (see, for instance, Kaup et al., 2016).

Evidence for the word-based mechanism is extensive. A number of behavioral studies have demonstrated that comprehenders reactivate spatial experiences when they encounter words whose referents are typically associated with an upper or lower vertical location (*implicit location words*; e.g., “satellite” vs. “grave”). For example, in a study by Lachmair et al. (2011), implicit location words were shown centered on the screen and in different font colors. Participants were asked to respond to the font color of the words by performing upward and downward arm movements. Crucially, even though the task did not require lexical access, response times were faster when the movement direction matched the typical vertical location of the implicit location word. The identical pattern of results was obtained in further studies using highly similar materials and experimental procedures (e.g., Ahlberg et al., 2018; Dudschig et al., 2012, 2014, 2015; Öttl et al., 2017; Schütt et al., 2022; Thornton et al., 2013; Vogt et al., 2019). In another study by Dunn et al. (2014), participants made lexical decisions on auditorily presented implicit location words, non-spatial words, and non-words. Concretely, they provided their decision by fixating a target located above or below the screen center. As it turned out, initiating saccadic eye movements was faster when the vertical location typically associated with the implicit location word matched the saccade direction (e.g., when participants performed an upward saccade to state that “moon” is a word; see also Dudschig et al., 2013). Interestingly, Ansorge et al. (2010) used simple German words referring to an upper or lower spatial position on the vertical axis (translated into English: “on top”; “above”; “upward”; “high”; “downward”; “deep”; “down”; “below”) as primes and targets in a masked priming paradigm. The task was to press a higher response key when the target referred to a higher spatial position and to press a lower response key when the target referred to a lower spatial position. The results revealed that response times were faster when prime and target words had the same spatial feature (e.g., this was true for the prime–target pair “above–high”). This clearly illustrates that even rather unconscious word processing can influence subsequent sensorimotor processing. Moreover, there is also evidence from neuroscience suggesting that word processing involves reactivating sensorimotor experiences. For instance, reading odor-related words (e.g., “cinnamon”; “garlic”; “jasmine”) compared with reading odor-neutral words (e.g., “coat”; “poker”; “glasses”) induced an elicited activation in the primary olfactory cortex (González et al., 2006). Similarly, reading action verbs associated with movements of the face, the arm, or the leg (e.g., to “lick”; “pick”; “kick”) evoked a somatotopic activation in motor and premotor brain areas that are related to actual movements of the tongue, the fingers, or the feet (Hauk et al., 2004; but see Miller et al., 2018).

In contrast, the situation is much less clear for sentence-based simulations conveying meaning beyond the word level. Even though there has been research using sentence-based materials and producing results usually considered as simulation effects, it is uncertain whether these results reflect specifically sentence-based and not merely word-based simulation processes. A prototypical example for this issue is the seminal work of Zwaan et al. (2002), which asked whether language comprehenders mentally simulate the shapes of mentioned entities. Participants read sentences referring to entities in specific locations that modulated the implied shape of the entity in question. For instance, reading the sentence “The ranger saw the eagle in the sky” should trigger the simulation of an eagle with outstretched wings, but reading the sentence “The ranger saw the eagle in its nest” should be more likely to trigger the simulation of an eagle with folded wings. After reading sentences like these, participants saw an image of the entity and decided whether it had been mentioned in the sentence. The results revealed faster responses when the shape implied by the sentence matched the shape depicted in the image. Even though these effects were in response to sentences, it is also plausible that they were driven by individual words within those sentences, irrespective of sentential meaning composition. For example, associations with the words “eagle” and “sky” might have produced the mental simulation of an eagle with outstretched wings, whereas encountering the words “eagle” and “nest” might have elicited the mental simulation of an eagle with folded wings. In line with this interpretation, Kaup et al. (2007) found comparable simulation effects when sentences included a negation marker (e.g., “The eagle was *not* in the sky/nest”). Response times were faster when the picture matched the situation that was negated (e.g., an eagle with outstretched/folded wings) than when the picture matched the situation that was actually conveyed by the sentential meaning (e.g., an eagle with folded/outstretched wings). In addition, participants have been found to react faster to picture probes showing specific entity shapes indicated by sets of content words presented in word lists (Kaup et al., 2012). In sum, this line of work shows that simulation effects in response to sentences may or may not be attributable to simulation processes beyond the word level.

This same confound applies to other influential work in the area. The action-sentence compatibility effect (ACE; Glenberg & Kaschak, 2002) is the observation that participants are faster to judge the sensibility of sentences when the direction of the response movement matches (vs. mismatches) the direction of the action described in the sentences (e.g., when reacting with a movement towards the body to sentences such as “Courtney handed you the notebook” or “Andy delivered the pizza to you” compared with sentences such as “You handed Courtney the notebook” or “You delivered the pizza to Andy”). This ACE has recently been found to be hard to replicate (Morey et al., 2022; Winter et al., 2022). But even if the effect is real, it does not necessarily reflect simulation processes regarding the sentential meaning as a whole. Rather, participants might be reactivating sensorimotor experiences related to those words mentioned at the end of the sentence (e.g., “to you” and “handed you”: movement towards the body; “to Andy” and “handed Courtney”: movement away from the body), immediately before engaging their own motor response. The same is true of a similar paradigm, in which participants read sentences describing clockwise or counterclockwise manual rotations (e.g., “Jenny screwed in the light bulb” vs. “Liza opened the pickle jar”) while turning a knob device clockwise or counterclockwise (e.g., Capuano et al., 2022; Claus, 2015; Zwaan & Taylor, 2006). Once again, ACEs obtained in the context of this paradigm could equally be explained in terms of word-based effects.

Finally, this issue also appears to apply to experiments conducted in the context of a set of studies addressing the activation of specific hand-action representations during language comprehension (e.g., Bub & Masson, 2010; Masson et al., 2013; Masson, Bub, & Newton-Taylor, 2008; Masson, Bub, & Warren, 2008). In general, these studies distinguish functional hand actions related to interacting with an object according to its common function (e.g., pulling the trigger of a water pistol) and volumetric hand actions related to picking up or holding an object. For instance, in one experiment by Bub and Masson (2010) that might be affected by the confound discussed here, participants were presented with context sentences implying a functional or a volumetric hand action (e.g., “David wrote with the pencil” vs. “Bert picked up the pencil”). After a short or long delay (300 vs. 750 ms), which was accompanied by an image of the target referent (here: a pencil), a cue appeared prompting participants to perform an unrelated hand action or the functional or volumetric hand action typically associated with the object referenced in the sentence. Irrespective of the sentence context, there was a priming effect on response latencies for functional and volumetric actions after the short delay. In contrast, after the long delay, a priming effect was only present when the sentence context and the hand action matched. This might suggest that participants created a hand-action representation reflecting the sentential meaning over the course of time. However, it is again conceivable that these effects resulted from single words included in the sentences. For example, associations with the words “wrote” and “pencil” could have caused a functional hand-action representation, whereas reading the words “picked up” and “pencil” might have evoked a volumetric hand-action representation. Consequently—just as in the case of the examples outlined previously—the obtained results can be attributed to either sentence-based or word-based simulation effects.

The most compelling evidence for sentence-based simulation effects comes from studies using grammatical modifications to sentences for driving changes in simulation effects. For instance, Taylor and Zwaan (2008) observed that the rotational ACE persisted when a postverbal adverb referred to the matching action (e.g., “He found a new light bulb which he screwed in rapidly”) but ended when the postverbal adverb addressed the acting individual (e.g., “On the shelf, he found a closed jar which he opened hungrily”). Similarly, Bergen and Wheeler (2010) found that progressive sentences (e.g., “Beverley is closing/opening the drawer”) induce an ACE, whereas perfect sentences (e.g., “Beverley closed/opened the drawer”) do not. Another line of work (Bergen et al., 2007) showed that verbs of upwards or downwards motion provoke simulation effects when combined with concrete nouns (e.g., “The cork rocketed”), but not when combined with abstract nouns (e.g., “The numbers rocketed”). Moreover, a study by Bidet-Ildei, Gimenes, Toussaint, Almecija, et al. (2017) revealed that sentence plausibility can affect the judgment about biological motions. The visual detection capacity for human actions displayed under point-light conditions was better when an auditorily presented sentence including a congruent action verb was plausible compared with implausible (e.g., “The neighbor is running in the garden” vs. “The garden is running in the neighbor”), suggesting that simulations were influenced by contextual aspects beyond the word level (for related work, see Bidet-Ildei et al., 2020; Bidet-Ildei, Gimenes, Toussaint, Beauprez, et al., 2017). In general, however, findings suggesting sentence-based simulations are few and in some cases rely on effects that are hard to replicate.

Taken together, there is little doubt that comprehenders indeed generate word-based simulations, whereas clear evidence in favor of sentence-based simulations is still sparse. Therefore, the first aim of the present research was to provide a new method for investigating whether comprehenders engage in creating mental simulations beyond the word level when processing sentential materials. To this end, we adapted the sentence–picture verification framework (see Zwaan et al., 2002), building sentential materials containing words that independently should equally well activate both entity shapes that match the final sentence meaning and entity shapes that do not. For instance, a sentence like “The egg was in the fridge as Mary ate” includes the word “egg”, denoting an object that can take on different shapes, such as intact in its shell (i.e., unpeeled) versus cracked open and peeled. The sentence by design comprises a word associated with each of these shapes—“fridge” with the intact egg and “ate” with the cracked and peeled egg. A sentence–picture compatibility effect to a sentence like this would therefore be unlikely to derive from lexical associations alone.

As a second-order question, we also interrogated the time course of simulation processes during sentence comprehension. If simulations are constructed on the basis of language structures larger than the word alone, then does this occur incrementally over the course of processing an utterance, or does it wait until the end of a sentence, manifesting as a sort of sentential wrap-up effect? The incrementality of sentential mental simulations is an issue that has barely been tackled. Available evidence stems from research investigating the modulation of the rotational ACE during sentence comprehension. For instance, in the study by Zwaan and Taylor (2006), participants turned a knob device clockwise or counterclockwise to read sentences describing manual rotations frame by frame in a self-paced manner (e.g., “He / realized / that / the music / was / too loud / so he / turned down / the / volume”). Interestingly, the authors found that the rotational ACE occurred when encountering the critical verb region referring to the manual rotation movement (e.g., “turned down”). This suggests that participants immediately created motor simulations and did not wait until the end of the sentence. In another line of work, Sato et al. (2013) made use of the verb-final word order of the Japanese language. In one of their experiments, they investigated whether comprehenders build specific object shape simulations even before reaching the verb at the end of a sentence. Participants were presented with sentences generating the expectation of a certain object shape prior to encountering the verb. For instance, an item paraphrased as “Mother put the shirt neatly in the drawer” was arranged in the typical Japanese word order: “Mother-NOM shirt-ACC drawer-LOC neatly put”. Crucially, reading the preverbal arguments could provide sufficiently constraining information for the comprehender to infer that the shape of the shirt was folded. To test for this early activation of scene-compatible object shape, participants responded to a picture probe *before* the verb appeared. The results showed faster responses when the shape implied by the preverbal phrase matched the depicted shape, indicating that detailed object shape simulations were created even though critical information was still missing. However—as the authors themselves noted—it is well possible that exposure to the picture probe itself prompted the participants to form detailed object shape simulations to perform the task, even if they would not have done so spontaneously during more naturalistic language processing. Thus, based on the few currently existing findings, it remains unknown whether language comprehenders routinely create incremental simulations during sentence processing. A second aim of our research therefore was to evaluate whether comprehenders construct mental simulations incrementally when reading sentences.

In order to investigate whether language comprehenders engage in forming sentence-based simulations and whether these are built in an incremental manner over the course of sentence processing, we presented participants with two kinds of sentences. The first were unambiguous sentences (e.g., “The egg was in the fridge as Mary ate”) comprising words that were associated with multiple possible shapes as described above. The second were manipulated versions of those same sentences, so-called garden-path sentences, which used the same words but were transitionally ambiguous (e.g., “As Mary ate the egg was in the fridge”). Typically, comprehenders interpret the first verb of such garden-path sentences as transitive (in the example: “Mary ate the egg”). If comprehenders formulate incremental simulations, they should thus activate an initial shape interpretation (e.g., a ready-to-eat egg). However, when arriving at the second verb, where they have to reanalyze the sentence, they should then activate the final sentence-based shape interpretation (e.g., an unpeeled egg in its shell). Previous research on incrementality of semantic and syntactic processing has found that the initial syntactic or semantic interpretation created during garden-path processing tends to linger after the sentence has been reanalyzed (Christianson et al., 2001; Patson et al., 2009; Slattery et al., 2013). So, if participants construct incremental simulations, there should be evidence of both object shape interpretations being activated at the end of the garden-path sentences. By contrast, the unambiguous control sentences should only evoke a single entity interpretation reflecting the sentence-based object shape interpretation (e.g., an unpeeled egg in its shell).

In each trial, participants read either a garden-path or a control sentence, followed by a picture probe displaying the target entity (e.g., a ready-to-eat egg vs. an unpeeled egg in its shell). If language comprehenders create mental simulations on the basis of the sentence as a whole, we should see faster picture-verification times when the picture probe matched the sentence-based interpretation of the target entity. However, if simulation effects are driven by independent word associations, then there should be no such difference—sentences like “The egg was in the fridge as Mary ate” include the same number of words consistent with each of the two possible depicted shapes of an egg. Moreover, if language comprehenders create sentence-level simulations incrementally, then the sentence–picture compatibility effect should be larger for unambiguous sentences than for garden-path sentences; since they will have representations corresponding to both shapes active at the end of the sentence in the garden-path condition, there should be a smaller difference between response times to the pictures or none at all.

## Experiment 1

### Method

#### Participants

We aimed to collect data from *N* = 96 participants through Amazon Mechanical Turk. All participants reported being right-handed native English speakers. They also declared normal or corrected-to-normal vision. Their ages ranged from 23 to 60 years (*M* = 38.21 years, *SD* = 9.60 years). There were 41 female and 55 male participants. In total, 19 additional participants completed the experiment, but were excluded and replaced due to an error rate higher than 25% in at least one experimental condition or on the filler trials. All participants gave informed consent and received $4.00 in return for participation. It took about 20 to 30 minutes to conduct the experiment. The study was approved by the Ethics Committee for Psychological Research at the University of Tübingen (Identifier: 2018_0831_132).

#### Apparatus and stimuli

We employed the open-source JavaScript library jsPsych (Version 6.1.0; de Leeuw, 2015) to implement a browser-based experiment. Participants were explicitly asked to use a laptop or a desktop computer for participating. They pressed the space bar to start trials and indicate that they had read and understood a sentence. The “d” key and the “k” key served as response keys in the sentence–picture verification task.

For experimental trials, we created 36 pairs of critical sentences. Each pair included one garden-path sentence (e.g., “While Amber hunted the turkey was on the table”; “As Zoe bathed the baby slept in the bed”; “While Ryan won the car was in poor condition”) and one matching unambiguous control sentence (e.g., “The turkey was on the table while Amber hunted”; “The baby slept in the bed as Zoe bathed”; “The car was in poor condition while Ryan won”). As described above, comprehenders tend to interpret the first verb of such garden-path sentences as transitive, constructing an initial interpretation of the target entity mentioned in the sentence (e.g., a living turkey; a baby in a bathtub; a car in brand-new condition; see, for instance, Christianson et al., 2001). Importantly, this initial interpretation corresponds to an intermediate processing step as comprehenders have to reanalyze the sentence when encountering the second verb, which should lead to creating a final sentence-based interpretation of the target entity (e.g., a ready-to-eat turkey; a dressed baby lying in the bed; a squalid car). Unambiguous control sentences, however, required comprehenders to form only a single interpretation of the target entity reflecting the sentence-based meaning (e.g., a ready-to-eat turkey; a dressed baby lying in the bed; a squalid car). As correctly answering experimental sentences always meant giving a “yes” response during the sentence–picture verification task, we also generated 36 filler sentences demanding a “no” response. Three fourths of the filler sentences followed the structure of unambiguous control sentences (e.g., “The scarf was in the washing machine as Bill knitted”); the remaining fourth of the filler sentences had the same structure as the garden-path sentences (e.g., “While Samuel ordered the fish swam upstream”). This reduced the proportion of sentences with garden-path structure participants encountered throughout the experiment. This in turn gave them fewer chances to learn the sentence structures and draw conclusions, reducing the likelihood that they would develop specific strategies with respect to garden-path processing (e.g., avoiding the initial entity interpretation). Four additional experimental sentences and four filler sentences were created for the practice session. Our sentential materials were partially adapted from or inspired by prior research (Christianson et al., 2001; Slattery et al., 2013; van Gompel et al., 2006).

**Fig. 1:**
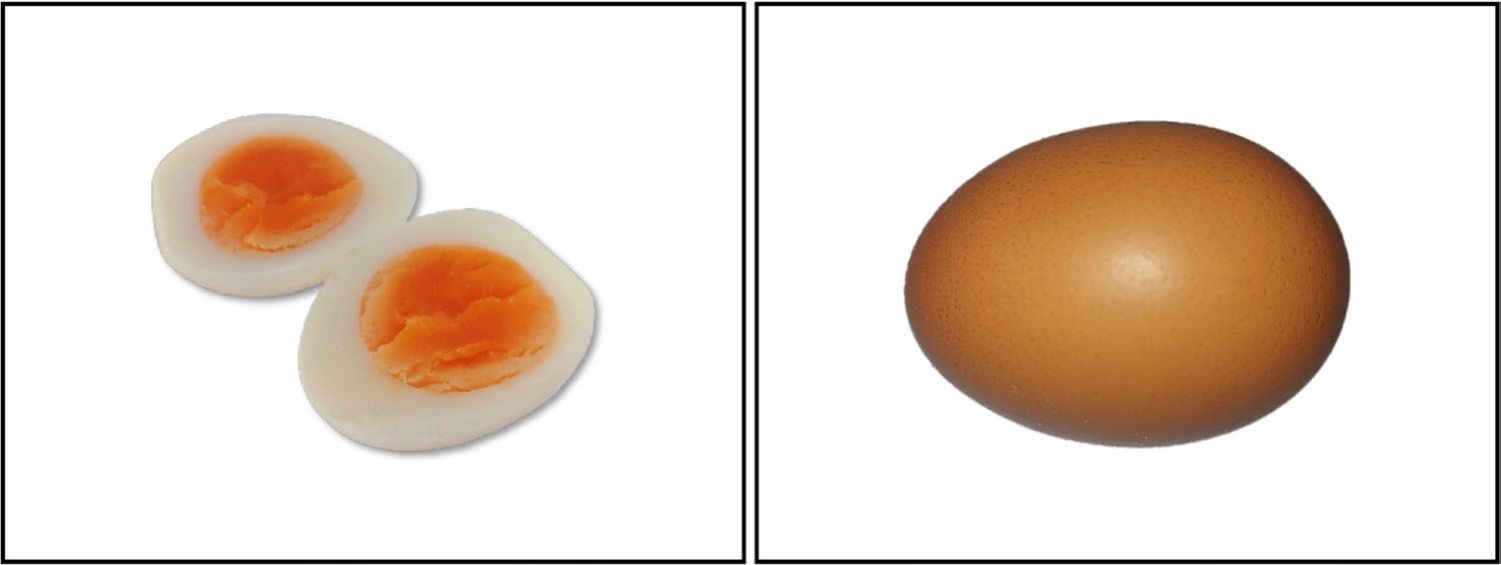
Example of a picture pair used in Experiments 1 and 2. The picture pair refers to the sentence pair comprising the garden-path sentence “As Mary ate the egg was in the fridge” and the unambiguous control sentence “The egg was in the fridge as Mary ate” (Experiment 1) or “As Mary ate the egg the butter was in the fridge” (Experiment 2), respectively. The ready-to-eat egg (picture on the left) showed the initial entity interpretation during garden-path processing, whereas the unpeeled egg (picture on the right) should illustrate the sentence-based entity interpretation. For the unambiguous control sentence, we expected the participants to generate a single entity interpretation corresponding to the sentence-based entity interpretation (Experiment 1: an unpeeled egg; Experiment 2: a ready-to-eat egg)

For each pair of critical sentences, there were two pictures showing the respective target entity mentioned in the sentences. One of the pictures depicted the entity in the shape implied by the initial interpretation that could be inferred during garden-path processing. The other picture displayed the entity in the shape corresponding to the sentence-based interpretation, which was always the same for both garden-path and unambiguous control sentences. For instance, for the garden-path sentence “While Amber hunted the turkey was on the table” and the corresponding unambiguous control sentence “The turkey was on the table while Amber hunted”, one picture showed a living turkey (initial entity interpretation during garden-path processing), whereas the other picture depicted a ready-to-eat turkey as served at Thanksgiving (sentence-based entity interpretation). A pretest ensured that the pictures referring to the target entity were comparable with respect to how clearly they depicted the entity irrespective of the shape (all *p*s > .05).^1^ In filler trials, we presented participants with pictures showing an entity not mentioned in the respective filler sentence. For example, the filler sentence “The scarf was in the washing machine as Bill knitted” was followed by a picture of green olives. Pictures were in color and scaled to a size of 768 (width) × 576 (height) pixels. Some example materials are given in Table 1 as well as in Fig. 1. For copyright reasons, we are not able to make the pictures publicly available. However, all sentential and pictorial materials will be made accessible upon scientific request (please contact the corresponding author).

**Table 1:** Examples of the sentential materials used in Experiments 1 and 2

Sentence type	Entity interpretation	
	Initial	Final
Garden-path		
1: As Mary ate the egg was in the fridge.	ready-to-eat egg	egg in its shell
2: While Edward painted the house was afire.	intact house	burning house
3: As Eve walked the dog lay on the ground.	walking dog	lying dog
4: While Miranda stirred the coffee was roasted.	cup of coffee	coffee beans
Control (Experiment 1)		
1: The egg was in the fridge as Mary ate.	not available	egg in its shell
2: The house was afire while Edward painted.	not available	burning house
3: The dog lay on the ground as Eve walked.	not available	lying dog
4: The coffee was roasted as Miranda stirred.	not available	coffee beans
Control (Experiment 2)		
1: As Mary ate the egg the butter was in the fridge.	not available	ready-to-eat egg
2: While Edward painted the house the forest was afire.	not available	intact house
3: As Eve walked the dog the cat lay on the ground.	not available	walking dog
4: While Miranda stirred the coffee the potato was roasted.	not available	cup of coffee

Comprehenders tend to interpret the first verb of garden-path sentences as transitive, resulting in an initial entity interpretation. However, when arriving at the second verb, the sentence must be reanalyzed, inducing the final sentence-based entity interpretation. By contrast, comprehenders should only create a single (sentence-based) entity interpretation in unambiguous control sentences

#### Procedure

We instructed our participants to participate in the experiment in an interference-free environment. Each trial started with the prompt “Please press the space bar to initiate the trial”. After pressing the space bar, a fixation cross (“+”; 800 ms) appeared centered on the screen. Then the fixation cross was replaced by a critical or a filler sentence. Participants were asked to read the sentence at a normal pace and to press the space bar. After this, a blank screen followed (500 ms), before participants were presented with the picture. Their task was to judge whether the depicted entity was mentioned in the previous sentence or not. Half of the participants pressed the “d” key for a “yes” response and the “k” key for a “no” response. For the other half of the participants, the response mapping was reversed. They were instructed to provide their response as fast as possible. During practice trials, participants received feedback regarding their response accuracy (“Correct!” vs. “Wrong!”; 1000 ms). The intertrial interval was 1500 ms. Initially, participants participated in a practice session. Subsequently, they performed three experimental blocks, each consisting of 12 critical and 12 filler trials. The conditions for each item (sentence type: garden-path vs. control sentence; picture type: compatible vs. incompatible with the sentence-based entity interpretation) were counterbalanced using four lists. Likewise, conditions were counterbalanced within the blocks. The order of trials was randomized. After each block, there was a self-paced break.

#### Design and data analysis

The experiment had a 2 × 2 within-subjects design, including the factors sentence type (garden-path vs. unambiguous control sentence) and picture type (compatible vs. incompatible with the sentence-based entity interpretation). Importantly, regarding the factor “picture type”, the level “incompatible with the sentence-based entity interpretation” reflected the initial entity interpretation during garden-path processing (i.e., this particular entity interpretation should not be formed during control sentences). The time period from the occurrence of the picture on the screen until pressing the response key (picture response time) served as dependent variable. All data and R analysis scripts are publicly available online (10.5281/zenodo.6504181).

We preprocessed and analyzed picture response times using the free statistical software R (Version 4.1.1). First, we removed filler trials and incorrectly answered critical trials. After this, extreme outliers were eliminated (picture response times shorter than 150 or longer than 3000 ms, respectively). Finally, to detect further outliers, we applied the two-step procedure proposed by Kaup et al. (2006). We transformed the picture response times of each participant to *z*-scores and discarded picture response times with a *z*-score that deviated more than two and a half standard deviations from the mean *z*-score of the respective item in the respective condition. In all, outlier exclusion reduced the data set by less than 4%. We made use of the R package lme4 (Version 1.1-27.1; Bates et al., 2015) to build a linear mixed model (see Baayen et al., 2008). Our model contained fixed effects for sentence type, picture type, and the interaction of both factors. In order to arrive at a suitable random effects structure, we referred to the data-driven model selection criterion introduced by Matuschek et al. (2017), which aims at balancing Type I error rate and power. When performing the procedure, however, we obtained warning messages indicating singular fits and convergence issues with respect to more complex models. Consequently, our model was finally restricted to include random intercepts for participants and items. For assessing the significance of the fixed effects, we employed the function *mixed* from the R package afex (Version 1.0-1; Singmann et al., 2021), which estimates mixed models based on lme4. We calculated *p* values through likelihood ratio tests (i.e., we chose the option “LRT” for the argument *method* within the function *mixed*)*.* Generally, this means that the goodness of fit of a model with a specific fixed effect and the goodness of fit of a model without this specific effect were compared by referring to the ratio of their likelihoods. In case of the function *mixed*, the complete model with all fixed effects under consideration must be entered; the function then automatically builds suitable reduced models and performs likelihood ratio tests to compute *p* values for all fixed effects included in the complete model.

We also evaluated reading times (i.e., the time period from the appearance of the sentence on the screen until pressing the space bar). Particularly, we were interested in whether reading times were modulated by the factor sentence type. For this purpose, we processed and analyzed reading times in the same way as picture response times, except for the following adaptations. First, we defined extreme outliers as reading times shorter than 500 or longer than 7000 ms, respectively. In total, removing outliers—including the two-step procedure suggested by Kaup et al. (2006)—reduced the data set by less than 7%. Second, the mixed model solely comprised a fixed effect for the factor sentence type. Again, we skipped some more complex models due to a singular fit when determining the random effects structure. The final model contained random intercepts for participants and items and by-item random slopes for sentence type.

## Results and discussion

**Fig. 2:**
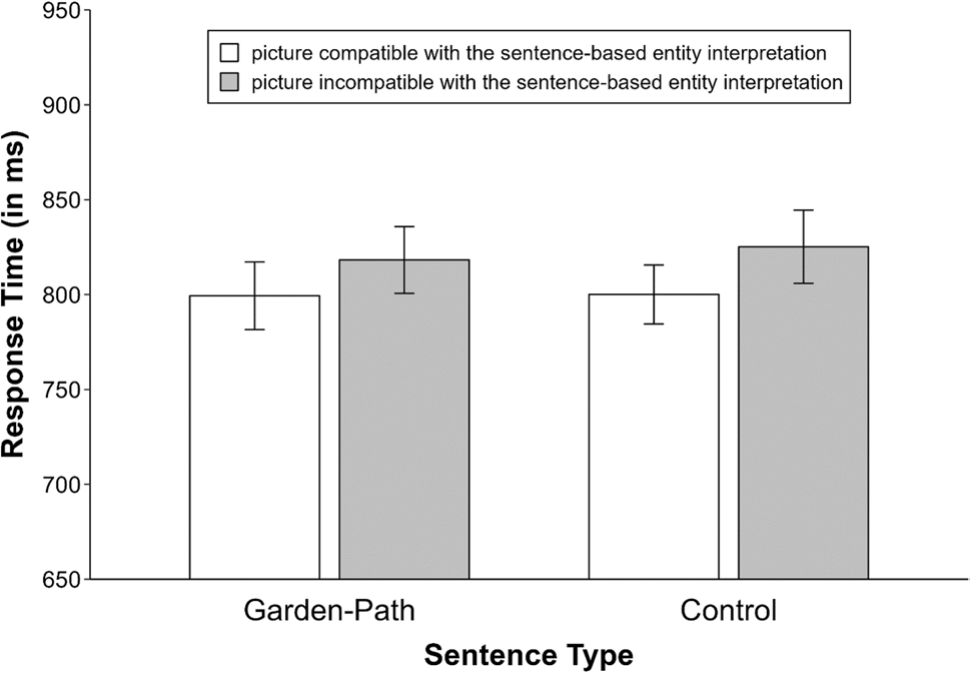
Mean response times for the sentence–picture verification task in Experiment 1. Error bars denote 95% within-subjects confidence intervals calculated as recommended by Morey (2008)

The data analysis revealed that there was a significant effect of sentence type on reading times, *χ*^2^(1) = 17.78, *p* < .001. As expected, participants needed more time to read garden-path sentences (*M* = 1812 ms) than unambiguous control sentences (*M* = 1677 ms). Figure 2 depicts the mean response times for the sentence–picture verification task as a function of sentence type and picture type. The effect of sentence type turned out not to be significant, *χ*^2^(1) = 0.80, *p =* .372. However, the results showed that there was a significant effect of picture type, *χ*^2^(1) = 9.42, *p* = .002, with participants responding faster when the picture probe matched (*M* = 800 ms) compared with mismatched (*M* = 822 ms) the sentence-based entity interpretation.^2^ This effect was not significantly modulated by sentence type, *χ*^2^(1) = 0.58, *p* = .446.

First, these results can be interpreted as evidence with respect to the validity of the experimental procedure. Specifically, reading times were slower for garden-path sentences than for control sentences. This most likely reflects the additional processing difficulties associated with garden-path sentences (e.g., Ferreira & Henderson, 1991; Frazier & Rayner, 1982; Pickering & Traxler, 1998). This provides indirect evidence that participants read the sentences for comprehension and indeed created an intermediate, incremental interpretation of some kind when facing garden-path sentences. Participants also responded faster to picture probes compatible with the sentence-based entity interpretation. Since our materials were explicitly constructed to be less prone to word-based effects, this suggests that participants indeed generated sentence-based simulations. As argued, this comes against a backdrop of quite sparse evidence in favor of sentence-based simulations.

However, it should be noted that limitations in the construction of experimental materials did not allow us to manipulate which entity shape interpretation and thus which picture of a target entity was associated with the sentence-based interpretation in an individual item. For instance, for the garden-path sentence “As Mary ate the egg was in the fridge” and the control sentence “The egg was in the fridge as Mary ate”, the sentence-based entity interpretation could not be varied and always corresponded to the unpeeled egg in its shell. Therefore—even though the pretest indicated that both pictures related to a target entity similarly clearly depicted this target entity—we cannot rule out the possibility that picture probes referring to the sentence-based shape interpretation were somehow preferred for the entities in question, and thus led to faster picture response times. Moreover, it remains possible that even if we included words in sentences aligned with each of the two entity shape interpretations, nevertheless these might have had unbalanced effects such that one shape was more consistent with the aggregate lexical associations of the sentence.

To address these limitations, we conducted a second experiment, adapting the materials in such a way that the sentence-based entity interpretation in control sentences was linked to the opposite shape and picture from the current experiment. Importantly, if the observed effect is due to comprehenders simulating the sentence-based meaning—and not an artefact of picture preference—the advantage for pictures compatible with the sentence-based entity interpretation should still occur in both garden-path sentences and control sentences. Since we did not observe evidence for the creation of incremental simulations during sentence comprehension in the present experiment (there was no significant interaction of picture type and sentence type), our material adaptions additionally aimed to create more fertile conditions for garden-path effects by making it more difficult to quickly identify this sentence type upon sentence presentation. Prior to starting data collection, we preregistered the experiment (https://aspredicted.org/tq6p9.pdf).

## Experiment 2

### Method

#### Participants

Based on the results of a pilot study, we conducted a simulation-based power analysis for the linear mixed model by using the R package mixedpower (Kumle et al., 2021). This revealed that we would need about 200 participants to reach a power of at least .80 for each of the fixed effects included in the model (sentence type; picture type; interaction of sentence type and picture type). We recruited participants via the crowdsourcing online labor marketplace Prolific. All participants (173 females, 27 males) reported themselves to be right-handed native English speakers. Their ages ranged between 18 and 55 years (*M* = 23.51 years, *SD* = 5.85 years). As in Experiment 1, we excluded and replaced participants with an error rate higher than 25% in at least one experimental condition or in filler trials. For this reason, there were seven additional participants who performed the experiment. All participants gave informed consent and received £4.00 in compensation for participation. It took about 20 to 30 minutes to finish the experiment. The study was approved by the Ethics Committee for Psychological Research at the University of Tübingen (Identifier: 2018_0831_132).

#### Apparatus and stimuli

Sentential materials were modified from Experiment 1. The garden-path sentence in each pair of critical sentences stayed exactly the same, but we replaced the unambiguous control sentences. Like the original control sentences, the newly introduced control sentences were transformed versions of the corresponding garden-path sentences. However, these were created by inserting an additional noun phrase referring to a task-irrelevant entity prior to the second verb of the garden-path sentence. For instance, the garden-path sentence “As Mary ate the egg was in the fridge” was transformed into the unambiguous control sentence “As Mary ate the egg *the butter* was in the fridge”. Thus, as in Experiment 1, understanding the unambiguous control sentences should involve forming a single entity interpretation fitting the sentence-based meaning (e.g., a ready-to-eat egg in the control sentence mentioned above). This time, however, the sentence-based entity interpretation always referred to the opposite shape and picture from Experiment 1. Furthermore, in Experiment 2, garden-path sentences and the newly introduced control sentences had very similar structure, which we hoped would make identifying the sentence type at hand and applying specific strategies (e.g., avoiding the initial entity shape interpretation during garden-path processing) less likely to occur. Filler and training sentences were adapted in an analogous manner. Table 1 provides further sample materials.

#### Procedure

The procedure was identical to that of Experiment 1.

#### Design and data analysis

The design was the same as in Experiment 1, except for the crucial fact that the sentence-based entity interpretation in control sentences reflected the opposite shape and picture from Experiment 1 (this was always identical to the initial entity interpretation during garden-path processing).^3^ All data and R scripts can be found online (10.5281/zenodo.6504181). Data preprocessing and data analysis fully followed the procedure employed in Experiment 1. Outlier elimination reduced the data set with respect to picture response times and reading times by less than 4%. When performing the method for determining an appropriate random effects structure for the mixed models, some more complex models were rejected due to convergence issues or a singular fit. Ultimately, the model for analyzing picture response times included random intercepts for participants and items and by-item random slopes for sentence type, picture type, and the interaction of sentence type and picture type. The model for reading times comprised random intercepts for participants and items as well as by-item random slopes for sentence type.

## Results and discussion

**Fig. 3:**
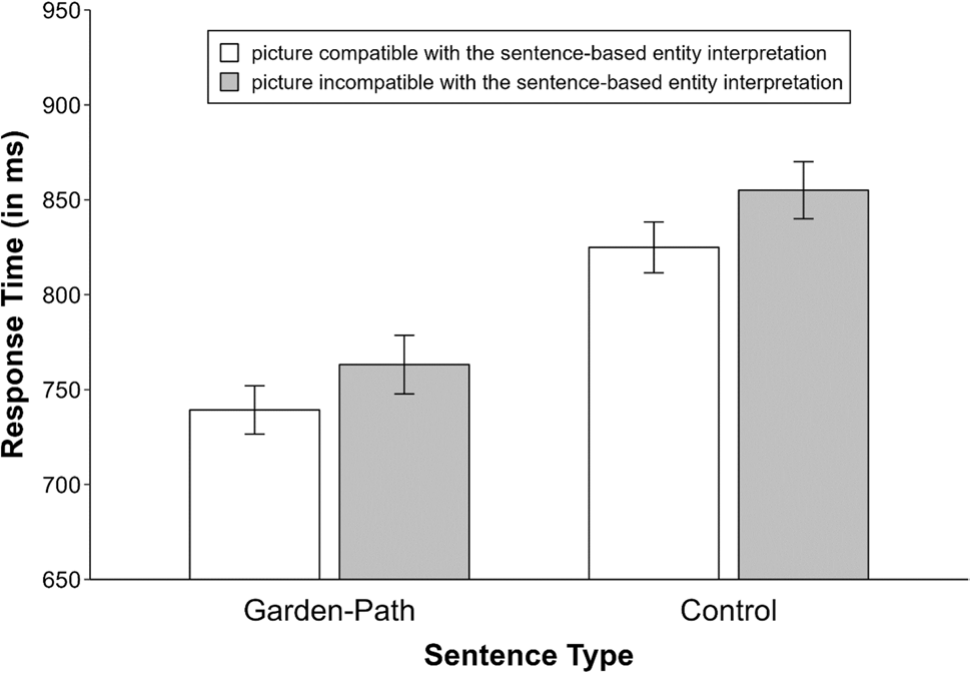
Mean response times for the sentence–picture verification task in Experiment 2. Error bars denote 95% within-subjects confidence intervals calculated as recommended by Morey (2008)

There again was a significant effect of sentence type on reading times, *χ*^2^(1) = 67.55, *p* < .001. This time, participants needed more time to read control sentences (*M* = 2322 ms) than garden-path sentences (*M* = 1946 ms), which is not surprising given the fact that control sentences were now longer due to the additional noun phrase. Figure 3 provides an overview of the mean response times in the sentence–picture verification task. The effect of sentence type was significant, *χ*^2^(1) = 35.64, *p* < .001. Participants responded faster to pictures after garden-path sentences (*M* = 751 ms) than after control sentences (*M* = 840 ms). More importantly, the effect of picture type was also significant, *χ*^2^(1) = 11.11, *p* < .001. Just as in Experiment 1, response times were faster when the picture probe matched (*M* = 781 ms) compared with mismatched (*M* = 808 ms) the sentence-based entity interpretation.^4^ Once again, this effect was not significantly modulated by sentence type, *χ*^2^(1) = 0.00, *p* = .946.

Thus, all critical effects remained as observed in the first experiment. Most importantly, participants again responded faster to picture probes compatible with the sentence-based entity interpretation than to picture probes incompatible with the sentence-based entity interpretation. Since the sentence-based entity interpretation in control sentences was related to the opposite shape from the first experiment, we can rule out effects of picture preference or aggregate word association bias. This reinforces the conclusion that participants indeed generated sentence-based simulations. In line with the results of the first experiment, there was no evidence suggesting that participants created incremental simulations during garden-path processing. However, this time, response times to picture probes as well as reading times were faster in trials with garden-path sentences than in trials with control sentences. This could be due to several reasons. First, the control sentences now included an additional noun phrase and were therefore longer than the garden-path sentences. Second, research stemming from the context of the discourse model approach indicates that establishing and accessing more referents produces longer processing times (e.g., Murphy, 1984). It is conceivable that this is the result of mentally simulating the additional referent. Third, encountering two referents one after another—such as in the control sentence “As Mary ate the egg the butter was in the fridge”—may have provoked a shift of the focus to the entity mentioned second (i.e., the butter; foreground), thus leaving the actual target entity shown on the picture (i.e., the egg) in the background (for a review on the use of situation models in research on language comprehension including foregrounding, see Zwaan & Radvansky, 1998).

## General discussion

Over the last few years, embodied accounts of cognition have become increasingly important in theories and empirical research on human language comprehension. Crucially, these approaches (e.g., Barsalou, 1999; Glenberg & Kaschak, 2002; Zwaan & Madden, 2005) propose that we conceive the meaning of language via mental simulations, which are created by means of activating and combining sensorimotor experiences related to the referents of the linguistic input (e.g., words, phrases, or idioms). As of yet, however, there has been little focus on the processes underlying meaning composition during embodied sentence comprehension. In fact, prior research largely leaves it unknown whether reported effects resulted from sentence-based simulations or simulations based on single words or bags of words. In two experiments, we aimed to overcome this issue. We also investigated whether language comprehenders create incremental simulations during sentence processing, an aspect that has also been treated in a very limited way in research on sentence comprehension.

We presented participants with a sentence–picture verification task. In each trial, their task was to decide whether the entity shown in the picture was mentioned in the sentence they had previously read. Importantly, the sentential materials consisted of garden-path sentences (e.g., “While Mary ate the egg was in the fridge”) and unambiguous control sentences (e.g., “The egg was in the fridge while Mary ate”). Both for garden-path sentences and control sentences, we expected participants to respond faster to a picture probe showing the final sentence-based entity shape interpretation (e.g., an unpeeled egg in its shell). Moreover—as the other picture probe always displayed the initial entity shape interpretation incrementally created during garden-path processing (e.g., a ready-to-eat-egg)—we also hypothesized that this effect should be less pronounced for garden-path sentences than for unambiguous control sentences.

In the first experiment, participants indeed responded significantly faster when the picture probe was compatible with the sentence-based entity shape interpretation. However, as this effect was not modulated by sentence type, we did not find any evidence indicating that comprehenders create incremental simulations during sentence comprehension. In the follow-up experiment, we intended to exclude the possibility that the observed effect simply occurred because there was a general preference for the pictures displaying the sentence-based entity interpretation. Thus, we adapted the materials in such a way that the sentence-based entity shape interpretation in control sentences was related to the opposite shape and picture than in the first experiment. Nevertheless, participants again responded significantly faster when the picture probe corresponded to the sentence-based entity shape interpretation. Just as in the first experiment, this effect did not vary as a function of sentence type.

Most importantly, these results clearly indicate that participants tended to create sentence-based mental simulations when facing both garden-path and unambiguous sentences. Compared with previous research that might suggest similar conclusions (e.g., Glenberg & Kaschak, 2002; Zwaan et al., 2002), the sentential materials of the current experiments were constructed to be less prone to word-based interpretations. By ruling out certain alternative explanations, the findings presented here enrich the empirical evidence that mental simulation in sentence comprehension is compositional.

As do many studies using similar methods, the current work leaves unresolved the issue of whether mental simulations are functionally implicated in understanding linguistic meaning. It remains conceivable that sentence-based mental simulations are only formed after an amodal symbolic meaning composition has taken place. In this case, mental simulations of sentential meaning would constitute a by-product of the language comprehension processes. Although functional relevance is clearly one of the most interesting and pressing open issues in the area of embodied language comprehension, there has been little behavioral research on this topic. In addition, the few existing studies on functional role have largely focused on word-based mental simulations (e.g., Shebani & Pulvermüller, 2013; Strozyk et al., 2019; Yee et al., 2013). Future research is needed that comes up with research methods suited to produce informative results regarding the functional relevance of sentence-based mental simulations.

However, even though our experiments were not explicitly designed to examine the functional role of sentence-based mental simulations, they still provide at least some preliminary insights into this issue. In both experiments, we found no evidence indicating that participants formed incremental simulations during the processing of garden-path sentences. This suggests that mental simulations might not functionally contribute to understanding the meaning of language—indeed, prior research (e.g., Christianson et al., 2001; Patson et al., 2009; Slattery et al., 2013) has convincingly shown that comprehenders build a representation of the initial (i.e., incremental) entity interpretation when processing the sort of garden-path sentences used in the here reported experiments. Thus, the absence of evidence for incremental simulations could propose that generating mental simulations is an optional by-product of language comprehension processes.

However, the observed pattern of results (i.e., no evidence for mental simulations of the initial entity interpretation during garden-path processing) is inconsistent with prior findings by Sato et al. (2013), who also examined incremental simulations of entity shapes. Critically—and in contrast to the current experiments—they used a sentence–picture verification task including a picture probe located in the middle of the sentence. On the one hand, it is clearly possible that this procedure prompted participants to form the object shape simulation in question and thus artificially induced their observations pointing towards the existence of incremental simulations. On the other hand, a direct approach like the one they adopted could be necessary to prevent detection problems due to the de-activation or overwriting of early, incremental simulations in the further course of reading. Interestingly, Hoeben Mannaert et al. (2019) recently provided some initial insights into this issue when investigating the dynamics of mental simulations *across several* sentences. They presented participants with short narratives of two or four sentences. Most importantly, these narratives differed in whether they implied a change in the shape of a target entity (e.g., change of shape: “The eagle was moving through the air. That evening the eagle was resting in its nest.”; constant shape: “The eagle was moving through the air. That evening the eagle was still moving through the air.”). Then, a picture of the target entity appeared, either matching or mismatching the final entity shape (e.g., an eagle with folded vs. outstretched wings), and participants judged whether the entity was mentioned in the narrative or not. The results showed that response times were significantly faster when the picture matched with the shape implied by the final sentence of the narrative. This effect was not modulated by shape condition (change of shape vs. constant shape), even though in trials with a change of shape an initial and a final simulation of the entity shape should have been created. The authors thus proposed that the simulation of the final entity shape may have replaced the simulation of the initial entity shape. Although this finding refers to the dynamics of mental simulations *across* sentences, similar processes could occur *within* sentences. By this reasoning, our participants may have created incremental simulations but did not retain them sufficiently at the critical picture probe following the sentence presentation, making it hard or even impossible to detect them. On the contrary, there is also research indicating that comprehenders are able to preserve mental simulations of the orientation and shape of an entity over longer periods of time (e.g., Pecher et al., 2009) and that incremental entity interpretations during garden-path processing linger after the sentence has been reanalyzed (e.g., Christianson et al., 2001; Patson et al., 2009; Slattery et al., 2013). Altogether, it currently seems to be premature and inappropriate to draw any definite conclusions regarding the existence or relevance of incremental simulations during sentence comprehension.

Finally, the lack of evidence in favor of incremental simulations could also result from participants employing strategies to avoid forming the initial entity interpretation during garden-path processing. In the first experiment, garden-path and control sentences differed structurally, which subjects could have learned to attend to in order to process garden-path sentences more efficiently. We tried to overcome this issue by limiting the number of sentences that followed the garden-path structure—three fourths of the fillers had similar structure to the unambiguous control sentences. The intent was to make it harder for participants to become accustomed to garden-path sentences and develop specific processing strategies. Moreover, in the second experiment, participants should have had a harder time discriminating garden-path and control sentences at first glance, since they were identical for roughly the first half. Nonetheless, the pattern of results was similar in both experiments. This seems to argue for the validity of the procedure and against artefacts due to specific processing strategies.

In general, the sentence–picture verification framework used in the current experiments constitutes an important and well-established standard paradigm in the research on embodied language comprehension. Numerous behavioral studies rely on such tasks to investigate which aspects of meaning comprehenders tend to simulate when they encounter written linguistic stimuli, including the shape, size, color, orientation, and visibility of objects described in a sentence (e.g., Connell, 2007; de Koning et al., 2017a, 2017b; Stanfield & Zwaan, 2001; Yaxley & Zwaan, 2007; Zwaan & Pecher, 2012; Zwaan et al., 2002). More recently, the sentence–picture verification task has also served as a tool for revealing dynamic changes of mental simulations across sentences (see Hoeben Mannaert et al., 2019). Nevertheless, it is reasonable to question whether the approach is sufficiently sensitive to detect incremental processing steps during sentence comprehension because it does not allow for a direct look at on-line processes (for more general criticism on the validity of the sentence–picture verification task, see Ostarek et al., 2019).

In sum, we found evidence indicating that comprehenders tend to build sentence-based mental simulations. Importantly, the sentential materials were constructed to be less sensitive to simulation effects resulting from single words or bags of words than materials used in previous studies. Certainly, it remains an important and unanswered question whether these sentence-based simulations are functionally relevant for language comprehension. Beyond that, the pattern of results found no evidence that participants created incremental simulations related to the initial entity shape interpretation during garden-path processing. However, it cannot be ruled out that this finding emerged from methodological characteristics of the experiments. Future research needs to test the stability of these results by means of modified paradigms.

In conclusion, our experiments provide clear evidence for the idea that simulation effects are not limited to the word level but pertain to sentential meaning as well. Hence, our findings confirm a core assumption of embodied views of human language comprehension, which—as of yet—has not received much conclusive support in the literature. Furthermore, the results suggest that mental simulations might be created globally after meaning composition has taken place.

## Data Availability

All data generated during this research can be found online (10.5281/zenodo.6504181). Experiment 2 was preregistered (https://aspredicted.org/tq6p9.pdf).

## Footnotes

1 For the pretest, we recruited another 40 participants via Amazon Mechanical Turk. In each trial, we initially displayed the word denoting one of the target entities (e.g., “turkey”). Then, we presented the picture related to one of the two entity shapes (e.g., a living turkey or a ready-to-eat turkey). The task was to judge whether the picture showed the entity the word referred to.

2 As there was virtually no difference in error rates (picture compatible with sentence-based entity interpretation: 2.20%; picture incompatible with sentence-based entity interpretation: 2.26%), we can rule out an explanation in terms of a speed-accuracy tradeoff.

3 Please note that we used the same levels for the factor “picture type” as in Experiment 1, even though other levels were mentioned in the preregistration for Experiment 2. We did so to be consistent across all experiments.

4 As participants barely produced erroneous responses and error rates did not differ significantly (picture compatible with sentence-based entity interpretation: 2.44%; picture incompatible with sentence-based entity interpretation: 3.03%), we can rule out a speed–accuracy tradeoff.

## Code availability

All R analysis scripts are publicly available and can be found at the same place as the data.
